# Relative Power Correlates With the Decoding Performance of Motor Imagery Both Across Time and Subjects

**DOI:** 10.3389/fnhum.2021.701091

**Published:** 2021-08-13

**Authors:** Qing Zhou, Jiafan Lin, Lin Yao, Yueming Wang, Yan Han, Kedi Xu

**Affiliations:** ^1^Key Laboratory of Biomedical Engineering of Education Ministry, Department of Biomedical Engineering, Qiushi Academy for Advanced Studies, Zhejiang University, Hangzhou, China; ^2^Zhejiang Lab, Hangzhou, China; ^3^Frontiers Science Center for Brain and Brain-Machine Integration, Zhejiang University, Hangzhou, China; ^4^The College of Computer Science and Technology, Zhejiang University, Hangzhou, China; ^5^Zhejiang Key Laboratory of Neuroelectronics and Brain Computer Interface Technology, Hangzhou, China

**Keywords:** relative power, brain rhythms, motor imagery, performance variation, electroencephalogram

## Abstract

One of the most significant challenges in the application of brain-computer interfaces (BCI) is the large performance variation, which often occurs over time or across users. Recent evidence suggests that the physiological states may explain this performance variation in BCI, however, the underlying neurophysiological mechanism is unclear. In this study, we conducted a seven-session motor-imagery (MI) experiment on 20 healthy subjects to investigate the neurophysiological mechanism on the performance variation. The classification accuracy was calculated offline by common spatial pattern (CSP) and support vector machine (SVM) algorithms to measure the MI performance of each subject and session. Relative Power (RP) values from different rhythms and task stages were used to reflect the physiological states and their correlation with the BCI performance was investigated. Results showed that the alpha band RP from the supplementary motor area (SMA) within a few seconds before MI was positively correlated with performance. Besides, the changes of RP between task and pre-task stage from theta, alpha, and gamma band were also found to be correlated with performance both across time and subjects. These findings reveal a neurophysiological manifestation of the performance variations, and would further provide a way to improve the BCI performance.

## Introduction

Recent evidence suggests that Motor Imagery (MI) based Brain-Computer Interface (BCI) has great promise in motor functional rehabilitation with stroke patients (Monge-Pereira et al., 2017). However, only a few MI-BCI systems have been applied so far for stroke patients as a standardized clinical treatment (Cervera et al., 2018; López-Larraz et al., 2018). One of the biggest obstacles to the wide adoption of BCI in stroke rehabilitation is the considerable variation in BCI performance. A previous study showed that about 30% of subjects failed to reach proficiency in using BCI systems over a standard training period (Blankertz et al., 2010). These subjects used to be described as “BCI illiterates” or “non-performers/responders/regulators.” Numerous studies have attempted to explain the causes or reasons for this phenomenon (Ahn and Jun, 2015). Prior researchers have shown how basic individual characteristics (e.g., gender, age, or lifestyle) may match BCI performance. A study reported that females and users who play musical instruments well are likely to be good BCI performers (Randolph, 2012). Besides, it was found that neuroanatomical features were also correlated with BCI performance. For example, the structural integrity and myelination quality of deep white matter structures were found to be positively correlated with individual performance (Halder et al., 2013). These basic physiological factors can be classified as stable traits that usually are difficult to change in a short time. On the other hand, some studies showed that psychological states (e.g., motivation, confidence, or frustration) were also associated with BCI performance (Nijboer et al., 2011). Besides, better spatial imagination abilities (e.g., kinesthetic imagination scores or mental rotation scores) may account for better MI-BCI performance (Vuckovic and Osuagwu, 2013; Jeunet et al., 2015). These psychological factors can be classified as fluctuating states of users, reflecting the differences in mental states. These studies indicated that regulating or learning mental states in a relatively short time may be a potential solution to improve the performance of “BCI illiteracy” users.

At present, there are still some difficulties in quantifying the psychological states or mental abilities objectively. It is widely accepted that neurophysiological signals [e.g., electroencephalography (EEG), functional Magnetic Resonance Imaging (fMRI), or magnetoencephalography (MEG)] can be used to infer psychological states and can be measured in real-time (Borghini et al., 2014). Hence the neurophysiological interpretation of the BCI performance variation has received increased attention in recent years. A decade ago, the power spectral density (PSD) of sensorimotor rhythms (SMR) from a 2-min resting state was proposed as a neurophysiological predictor of MI performance (Blankertz et al., 2010). From previous studies, it is well known that SMR power decreased during MI, which is defined as event-related desynchronization (ERD). A possible explanation is that higher SMR from the resting state yields a larger decrease in MI tasks, which may result in better performance. After that, a study reported that higher frontal theta and lower posterior alpha-band powers during a few seconds before MI can represent the higher attentional level of the users, and thus may enhance the BCI performance (Bamdadian et al., 2014). Whereas another study focused on relative power level (RPL) of resting-state suggested that higher theta and lower alpha band RPL may indicate lower performance (Ahn et al., 2013). These plausible conflicting results here may due to the difference in experimental paradigms and parameters (e.g., signal segments, feature selections, or brain regions), which results in the difficulty to compare and reproduce previous findings.

On the other side, several intra-subject studies investigated the neurophysiological factors affecting performance over time. Trial-wise studies found that pre-cue SMR and gamma-band oscillations were positively correlated with performance within a day (Grosse-Wentrup and Schölkopf, 2012; Maeder et al., 2012). Another session-wise study on 13 stroke patients suggested that the pre-cue relative beta band power, as a mental fatigue index, was positively correlated with MI performance over 6 weeks (Foong et al., 2020). These findings suggest that some of the neurophysiological factors may reflect the changes of mental states in the time domain and could be further used for monitoring and updating the system according to the expected mental state.

In addition to the resting-state factors, the association between performance and the physiological state changed from pre-task to during-task stage was also considered an important influencing factor for BCI performance. A study found that on-task changes of Mahalanobis distance of theta power were negatively correlated with MI performance across subjects (Trambaiolli et al., 2019). They suggested that higher bilateral theta activity, which leads to lower Mahalanobis distance, may represent greater attention or working memory load and thus results in greater BCI performance. Another study found a negative correlation between BCI scores and relative on-task changes in both high alpha and low beta powers defined by Individual Alpha Frequency (IAF) across four sessions (Corsi et al., 2020). This result is reasonable that the higher decoding BCI scores were associated with a stronger decrease of SMR power during MI, i.e., a larger ERD.

By far, the neurophysiological factors of performance variation have not been systematically investigated. Most studies concentrated on a single perspective across subjects or time and some results were limited by relatively small sample size. Besides, the neurophysiological factors proposed before hardly achieved the expected results in replicate experiments (Jeunet et al., 2015; Zhang et al., 2015). Therefore, the purpose of our study was to explore: (1) the similarities and differences in performance variation from inter-subject and inter-session analysis. (2) the correlation between neurophysiological signals and MI performance across subjects and time. (3) whether there is a relatively stable neurophysiological factor to indicate the BCI performance.

## Materials and Methods

### Subjects and Experimental Protocol

Twenty healthy subjects (11 males, mean age: 23.2 ± 1.47 years, range 21–27 years, all right-handed) participated in the study. Four of them had participated in MI studies before. None of the participants reported a history of psychiatric or neurological disorders. The study was designed and conducted according to the Declaration of Helsinki and was approved by the Human Research Ethics Committee of The Second Affiliated Hospital of Zhejiang University School of Medicine. All subjects were asked to read and sign an informed consent form before the experiment and received financial compensation after the experiment for their time and effort.

**FIGURE 1:**
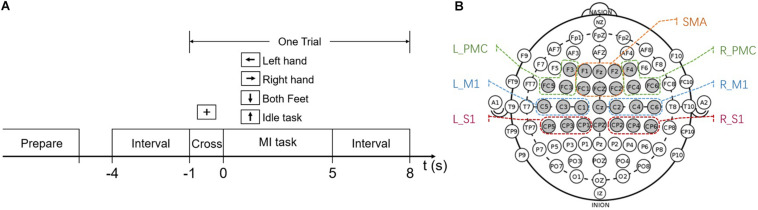
**(A)** MI-BCI experiment paradigm. **(B)** Chosen electrodes (gray) in the international 10–20 system. The electrodes are artificially grouped into different brain regions according to their location and marked in the figure. SMA, supplementary motor area; L_PMC, left premotor cortex; R_PMC, right premotor cortex; L_M1, left primary motor cortex; R_M1, right primary motor cortex; L_S1, left primary sensory cortex; R_S1, right primary sensory cortex.

The subjects were asked to participate in seven sessions across 2 weeks, once every 2 days. Each session lasted around 40 min and was organized into 6 runs. Subjects have a short rest between runs. During each run, subjects performed 40 trials (4 different MI-tasks, 10 trials per task, presented in random order), with each trial lasting 9 s (Figure 1A). During the experiment, subjects sat in a comfortable chair in front of a computer screen and were instructed to relax their arms, minimize any physical movement or eye blinking throughout the EEG recording process. At the beginning of a trial, a fixation cross appeared on the screen and stayed for 1 s. After that, a cue in the form of an arrow appeared to inform the subjects to start performing MI tasks. The arrow pointed either to the left, right, down, or up, and subjects were asked to perform MI tasks of the left hand, right hand, both feet, and the idle task, respectively. Each task lasted for 5 s. During the idle task, subjects were instructed to relax and think about nothing. After the task, an inter-trial interval of 3 s was followed. No feedback was provided for the subjects during the experiment.

### EEG Recording and Pre-processing

EEG recording was performed using a 64-channel Synamps2 system (Neuroscan, Inc.) with a sampling frequency of 500 Hz. Twenty-six EEG scalp electrodes were prior-selected according to the international 10–20 system, as seen in Figure 1B. The reference was on the top of the head, and the ground was on the medial frontal of the head. The horizontal electrooculogram (EOG) and vertical EOG were recorded using the same system. A band-pass filter between 0.5 and 100 Hz and a notch filter of 50 Hz was applied directly to the amplifier.

The obtained EEG signals were then pre-processed to reduce biological artifacts such as eye movements, blinking, heart, and muscular activities. The Independent Component Analysis (ICA) was applied to eliminate interference from eye movement and blinks (Vigario et al., 2000). In this study, the function was implemented through the MNE-Python package (Gramfort et al., 2013; Ablin et al., 2018). The dedicated EOG sensors were used as a “pattern” to check the Independent Components (ICs) against, any ICs that match the EOG pattern were automatically marked and excluded. In addition, a single-channel method, EEMD-CCA, was used to reduce the muscle noise contamination in the EEG, which is a combination of Ensemble Empirical Mode Decomposition (EEMD) and Canonical Correlation Analysis (CCA) (Chen et al., 2016; Foong et al., 2020). Single-channel EEG signals were decomposed into multiple Intrinsic Mode Functions (IMFs) using EEMD. Then, muscle artifact components were isolated by CCA due to the low autocorrelation (Clercq et al., 2006).

### BCI Performance Analysis

After pre-processing, the classification result was analyzed offline as a measurement of BCI performance. First, the EEG signals were band-pass filtered by linear phase Finite Impulse Response (FIR) filter between 8 and 30 Hz. During each trial, signals from 0.5 to 4.5 s after task onset were extracted and split into 4 × 1 s epochs. EEG epochs were then spatially filtered using the One Versus Rest (OVR) Common Spatial Pattern (CSP) algorithm (Ang et al., 2012). The CSP algorithm aimed at finding spatial filters such that the band power of the spatially filtered EEG signals was maximally different between the two classes. For multi-class filtering, the OVR algorithm computed the CSP features that discriminate each class from the rest. After that, these features were fed into the Support Vector Machine (SVM) with a regularization parameter C of 0.8 and a Radial Basis Function (RBF) kernel to generate subject-specific models, which was implemented through the python package Scikit-learn (Pedregosa et al., 2011). A 10-fold cross-validation (CV) procedure was performed to validate the results.

The classification accuracy and macro-averaged F-score were used in this study to evaluate the performance of each subject in each session (Metz, 1978; Sasaki, 2007). In multi-class problems, macro-averaged F-score exhibits more robust than accuracy for performance assessment (Sokolova and Lapalme, 2009). The accuracy and macro-averaged F-score were calculated as follows:


(1) $$\text{Accuracy}=\frac{\text{TP}+\text{TN}}{\text{TP}+\text{TN}+\text{FP}+\text{FN}}$$

(2) $$\text{Precision}=\frac{\text{TP}}{\text{TP}+\text{FP}}$$

(3) $$\text{Recall}=\frac{\text{TP}}{\text{TP}+\text{FN}}$$

(4) $$F\mathrm{score}=2\times \frac{\text{Precision}\times \text{Recall}}{\left(\text{Precision}+\text{Recall}\right)}$$

where TP was the number of true positives, TN was the number of true negatives, FP was the number of false positives, and FN was the number of false negatives. For the macro-averaged results, the precision and recall were calculated independently for each label and then the average was taken.

### Relative Power Analysis

The EEG powers of each band were different across the subjects, thereby making it difficult to see a pattern at the group level. The Relative Power (RP), which indicates the ratio of the power of a frequency band to the total band power, could be used to reduce this problem (Ahn et al., 2013). In this study, RP was computed as follows:

First, the EEG segments from –4 to 5 s were extracted for each trial. To investigate the changes of RP in the time domain, each trial was split into pre-task stage (–3 to 1 s), cross stage (–1 to 0 s) and task stage (0.5–4.5 s) (see Figure 1A). The signals lasting 0.5 s at the beginning and end of tasks were discarded. The trials in the task stage were further split by the type of task. Second, the frequency bands ranged from theta (4–8 Hz), alpha (8–13 Hz), beta (13–30 Hz), and low gamma (30–50 Hz) were selected for spectral power analysis according to previous studies (Ahn et al., 2013). Welch’s method with a 500 ms Hamming window and no overlapping was used to calculate power spectral density (PSD), which is one of the most used spectral estimation techniques to date (Welch, 1976; Proakis and Manolakis, 1996). The absolute band power is equal to the area under the PSD curve and calculated by the integration method. After that, the RP of each band was obtained by dividing the absolute power by its total absolute power in 4–50 Hz. Then RP was averaged over 6 runs for each subject and session.

### Statistical Analysis

*T*-test was applied to statistically analyze the significance of RP difference for group analysis. Spearman–rank correlation and repeated measures correlation (Bakdash and Marusich, 2017) were performed to analyze the correlation between RP and the performance from subject and session aspects separately. A false discovery rate (FDR) of α = 0.05 was used to correct the significance level for multiple comparisons. The statistical and correlational methods used in this paper were implemented by the python package Pingouin (Vallat, 2018).

## Results

### MI Performance Across Subjects and Time

After the experiment, we first computed the offline classification accuracy and macro-F score to evaluate performance for each subject and session. The classification was mainly focused on the three-class classification of MI tasks (i.e., left hand, right hand, and both feet). The average accuracy for all the subjects and sessions was 50.43 ± 10.49%, and the macro-F score was 47.92 ± 10.45%. The across-subject accuracy ranged from 39.95 ± 1.82% to 73.49 ± 5.64% and the macro-F score from 37.09 ± 1.33% to 71.79 ± 6.89%. Besides, binary classification of the dominant-hand MI task (i.e., right hand) and the idle task was also investigated. The average accuracy was 68.95 ± 10.54%, and the macro-F score was 66.62 ± 10.93%. The across-subject accuracy ranged from 57.36 ± 3.55 to 90.03 ± 6.92% and macro-F score from 55.71 ± 3.24 to 89.14 ± 6.02%. The results showed that all of the subjects obtained performances higher than the random level of 33.33% for three-class classification and 50% for binary classification. The classification accuracy was further chosen for evaluating MI performance in the following because the accuracy and macro-F score performed consistently in this study.

**FIGURE 2:**
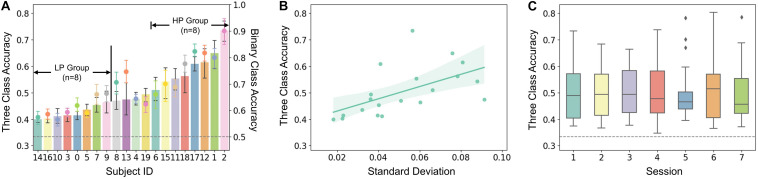
**(A)** Averaged accuracy of each subject. The histogram and dot plot represent three-class and binary class classification accuracy, respectively. The random levels of 33.33 and 50% are aligned and shown by a gray dotted line. Subjects were sorted and then assigned to the High Performance (HP, *n* = 8) and Low Performance (LP, *n* = 8) group according to the three-class accuracy performance. **(B)** Correlation between SD and three-class accuracy (*r* = 0.56, *p* = 0.01). Each dot represents a subject. **(C)** Averaged three-class accuracy of each session.

The three-class classification accuracy of each subject was plotted as columnar and binary classification accuracy as points in Figure 2A. According to the general performance, subjects were separated into the high-performance group (HP, *n* = 8) and low-performance group (LP, *n* = 8), which is also marked in Figure 2A, and the other four middle-performance subjects (id: 4, 8, 13, 19) were excluded. A positive correlation was found between averaged performance across sessions and its standard deviation (SD) (see Figure 2B, *r* = 0.735, *p* < 0.001). No significant performance increase was observed across sessions as shown in Figure 2C.

### Relative Power Difference Between High and Low Performance Group

**FIGURE 3:**
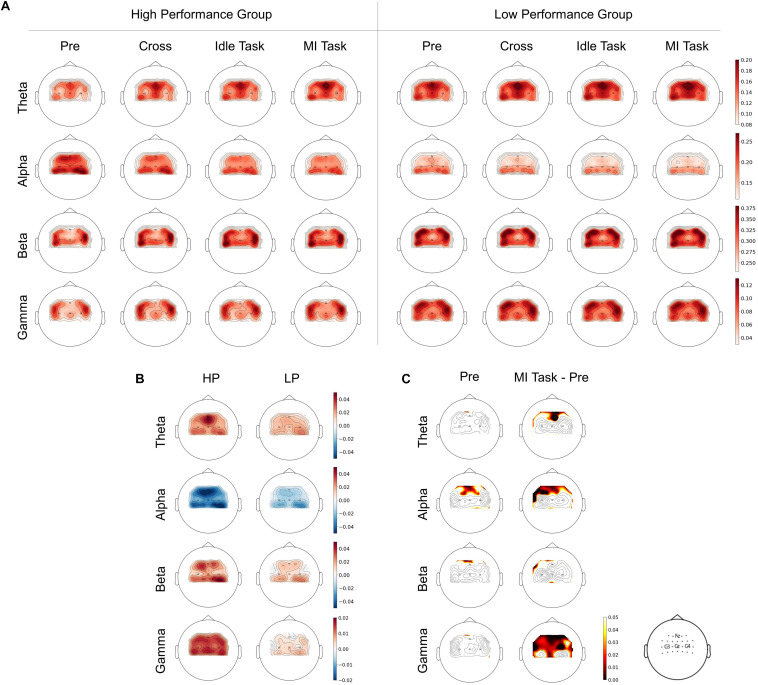
**(A)** Spatial distributions of RP between HP and LP group over four frequency bands and stages. **(B)** The difference between RP from MI task and pre-task stage. **(C)** The significant difference of *T*-test between groups from the pre-task stage and on-task changes (ΔRP).

To examine the relationship between RP changes and performance in the time domain, topographical images of RP were plotted as shown in Figure 3A. RP from four different stages (i.e., pre-task, cross, idle task, and MI task) and four frequency bands (i.e., theta, alpha, beta, and low gamma) were calculated in both groups. MI task stage was obtained by averaging three types of MI tasks (i.e., left hand, right hand, and both feet). As shown in Figure 3A, there was an observed difference between HP and LP groups during the pre-task stage. Specifically, the alpha band RP from the HP group was relatively higher than the LP group, whereas theta and gamma band RP were relatively lower. Besides, there are clear tendencies of decreasing from alpha band RP and increasing from theta and gamma band RP in the HP group during the process from the pre-task stage to the MI task stage, while no obvious trend was observed in the LP group.

To better demonstrate the RP changes during the process, the difference of RP between the MI task and the pre-task stage was calculated as ΔRP (see Figure 3B). After that, a *t*-test was applied to statistically analyze the significant difference between groups from the pre-task stage and on-task changes (ΔRP) (see Figure 3C). In the pre-task stage, alpha band RP from the HP group was significantly higher and mainly in the supplementary motor area (SMA), whereas no significant difference was found in the other three bands. For on-task changes, alpha band ΔRP was found significantly lower in the HP group mainly in the SMA and left premotor cortex (PMC). Whereas theta band ΔRP of the HP group was significantly higher in SMA, and gamma band was also significantly higher, mainly in SMA, bilateral PMC and primary motor cortex (M1), whereas no significant difference was found in beta bands.

### Correlations Between RP Factors and the MI Performance

**FIGURE 4:**
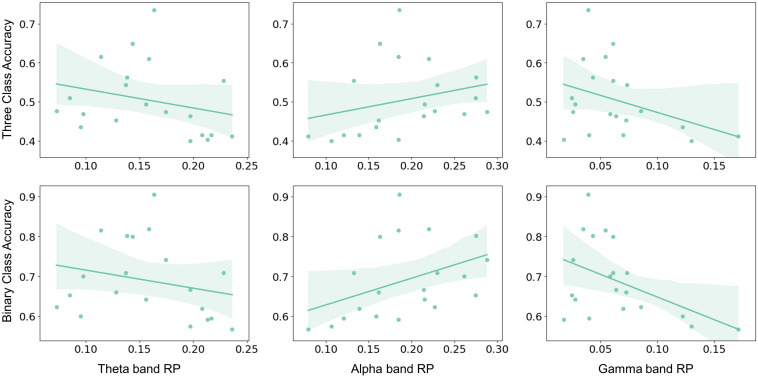
The Spearman–rank correlation between pre-task RP and performance across subjects. Alpha band RP was positively correlated with three-class classification accuracy (*r* = 0.484, *p* < 0.05) and binary-class classification accuracy (*r* = 0.546, *p* < 0.05). Whereas no statistical significance was found in the correlation between theta and gamma band RP and three-class classification accuracy (theta: *r* = –0.376, *p* = 0.102; gamma: *r* = –0.325, *p* = 0.162) or binary-class classification accuracy (theta: *r* = –0.284, *p* = 0.224; gamma: *r* = –0.369, *p* = 0.109).

**FIGURE 5:**
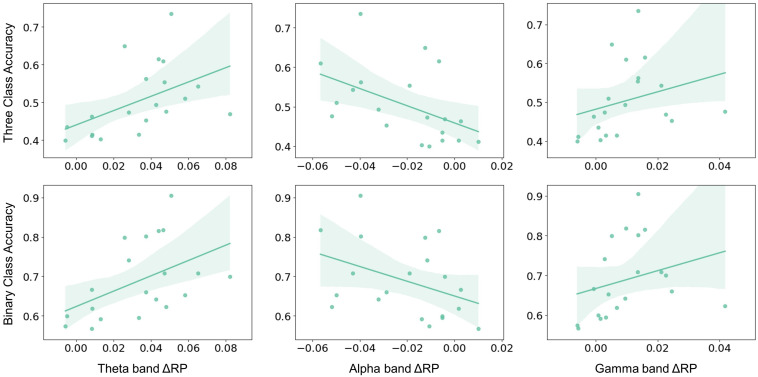
The Spearman–rank correlation between ΔRP and performance across subjects. Alpha band ΔRP was negatively correlated with three-class classification accuracy (*r* = –0.564, *p* = 0.01) and binary-class classification accuracy (*r* = –0.431, *p* = 0.058). Whereas theta and gamma band ΔRP were positively correlated with three-class classification accuracy (theta: *r* = 0.615, *p* < 0.01; gamma: *r* = 0.549, *p* < 0.05) and binary classification accuracy (theta: *r* = 0.54, *p* < 0.05; gamma: *r* = 0.528, *p* < 0.05).

The aforementioned significant frequency bands (i.e., theta, alpha, and gamma) from corresponding brain regions were selected as RP factors to further investigate to explain MI performance variation. The RP values were obtained by averaging the electrodes of the corresponding brain regions, as shown in Figure 1B. The MI performance was measured by three-class classification (i.e., left hand, right hand, and both feet) and binary-class classification (i.e., right hand and idle task) accuracy. Spearman-rank correlation was applied from both the pre-task stage and on-task changes. The result from the pre-task stage was shown in Figure 4. The alpha-band RP was found to be significantly and positively correlated with MI performance. Although a negative correlation was observed between performance and RP from theta and gamma band, it failed to reach statistical significance. For the results from on-task changes as seen in Figure 5, significant correlations were found between ΔRP and performance. The alpha band ΔRP was negatively correlated with performance, whereas theta and gamma band ΔRP were positively correlated with performance.

**FIGURE 6:**
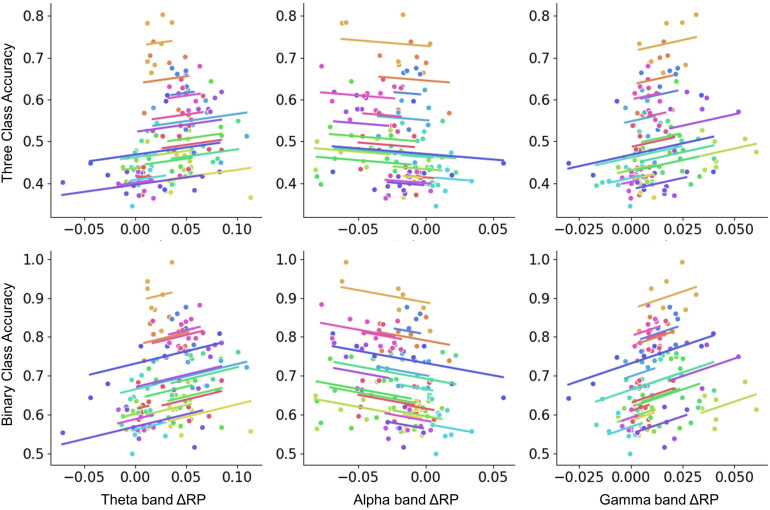
The repeated-measures correlation between ΔRP and performance across sessions. Colors identify the values obtained for the same subject across sessions. Binary-class classification accuracy was negatively correlated with alpha band ΔRP factor (*r* = –0.237, *p* < 0.001) and positively correlated with theta and gamma band ΔRP factors (theta: *r* = 0.277, *p* < 0.01; gamma: *r* = 0.348, *p* < 0.001). Whereas no significant correlation was found in three-class classification.

To investigate whether RP is correlated with performance across sessions, the repeated-measures correlation analysis that considered the time-domain nature of data was performed. From the pre-task stage, no significant correlation was found. From on-task changes, similar results were obtained when calculating the correlation between performance and ΔRP. As seen in Figure 6, theta and gamma band ΔRP were positively correlated with binary-class classification accuracy, while alpha band ΔRP was negatively correlated with binary-class classification accuracy. However, there was no significant correlation between ΔRP and three-class classification accuracy. We assumed that the correlation between ΔRP and performance was related to the type of task.

Moreover, the correlations between ΔRP and the classification accuracy from 11 combinations of four types of tasks (i.e., 6 for binary-class, 4 for three-class, and 1 for four-class) were analyzed. Table 1 presents the correlation coefficient (CC) and corresponding significance level from inter-subject and inter-session aspects, respectively. A false discovery rate (FDR) of α = 0.05 was used here to corrected the significance level. As seen from Table 1, ΔRP factors were significantly correlated with almost all combinations of classification results from the inter-subject aspect. In contrast, from the inter-session aspect, only the accuracies of classification containing idle tasks were found to be significantly correlated with ΔRP factors, especially from alpha and gamma bands.

**TABLE 1:** The correlation between ΔRP and performance across subjects and sessions.

	Intra-subject			Intra-subject		
Task	Theta	Alpha	Gamma	Theta	Alpha	Gamma
L-R	0.370	–0.496*	0.311	0.087	–0.073	0.161
L-F	0.648**	–0.568*	0.550*	0.045	–0.056	0.086
R-F	0.635**	–0.522*	0.577*	0.030	0.017	0.078
L-I	0.532*	–0.421	0.498*	0.223	–0.217*	0.294**
R-I	0.540*	–0.431	0.528*	0.277*	–0.237*	0.348**
F-I	0.627**	–0.466*	0.478*	0.138	–0.250*	0.260**
L-R-F	0.615**	–0.564*	0.549*	0.133	–0.089	0.187
L-F-I	0.660**	–0.512*	0.562*	0.161	–0.211*	0.287**
R-F-I	0.611**	–0.492*	0.544*	0.145	–0.176	0.219*
L-R-I	0.595**	–0.487*	0.490*	0.209	–0.235*	0.341**
L-R-F-I	0.651**	–0.541*	0.556*	0.208	–0.212*	0.313**

The first column represents the combination of tasks from which the classification accuracy was calculated. The inter-subject columns represent the Spearman–rank correlation coefficient between ΔRP and performance across subjects, and intra-subject columns represent the repeated-measures correlation coefficient between ΔRP and performance across sessions. The p-value was corrected by a false discovery rate. L, left hand MI; R, right hand MI; F, both feet MI; I, Idle task; *p < 0.05; **p < 0.01.

## Discussion

As mentioned in the literature review, performance variation has been an obstacle that degrades the reliability of BCI systems (Ahn and Jun, 2015). The subject who failed to reach a criterion level of performance in BCI tasks used to be labeled as “BCI illiterates.” Currently, some researchers are tending to adjust the former beliefs. They suggested that “BCI illiteracy” relies on the flawed assumption that users possess physiological or functional traits that prevent proficient performance during BCI use (Thompson, 2019). Moreover, no certain criteria were determined when identifying “BCI illiterates.” Consistent with previous studies (Blankertz et al., 2010), the performance of subjects in this study was found to be evenly distributed (see Figure 2A). Hence the proportion of “BCI illiterates” was largely determined by the criterion level which may be selected relatively arbitrarily. Therefore, the result of labeling users as “BCI illiterates” should be interpreted with caution. Besides, one of the results in this study indicated that higher performers seemed to be more variable (see Figure 2B), which was also reported in previous researches (Maeder et al., 2012). This may increase the difficulty of BCI applications and highlight the importance of investigating performance variation.

In reviewing the literature, the neurophysiological signal is considered a valid target to understand how BCI performance varies. However, no consistent conclusion was drawn on the proposed indicators and their relationships with performance. The relative power (RP) has been considered an important parameter for analyzing EEG during cognitive tasks, which have lower inter-subject variability and may be more reliable than absolute power (AP) (Nuwer, 1988; Harmonya et al., 1993). Other researchers suggested that AP and RP yield complementary information (Leuchter et al., 1993). Therefore, RP was selected here as a neurophysiological indicator to examine the relationship between brain rhythms and MI performance. As seen in Figure 3, PR differences of theta, alpha, and gamma band were observed between HP and LP groups during the process. Notably, it is well known that ERD was distinguished among different MI tasks. However, no significant RP difference was found among the three types of MI tasks, which is consistent with previous studies (Ahn et al., 2013). This result may be explained by the fact that the RP calculating in this study was normalized by total power, which largely diminished the ERD differences among tasks. Hence the RP value of the MI task here was obtained by averaging three types of MI tasks for further analysis.

In previous findings, higher alpha band PSD in the resting state has been found to correlate with better performance (Blankertz et al., 2010). In another cognitive performance study, alpha band power in the resting state was positively correlated with attention-span scores (Mahjoory et al., 2019). Hence the higher alpha band in the resting state may represent the potential larger ERD in the MI task as well as the higher attentional level of subjects. Although it is reasonable, the predictor of alpha-band power failed to reach the expected results in several similar experiments (Jeunet et al., 2015; Zhang et al., 2015). Since the band powers may vary from person to person, RP could be used to normalize features. In this study, the results of correlation analysis indicate that alpha band RP over SMA from a few seconds before MI was positively correlated with the BCI performance (see Figure 4). Similar results were obtained in previous studies (Ahn et al., 2013; Kwon et al., 2020). In addition, the alpha-band RP from the resting state was proposed before as an index of tracking cognitive function. Studies found that people with amnestic Mild Cognitive Impairment (aMCI) or Subjective Cognitive Decline (SCD) had lower alpha band RP compared with the control group (Bian et al., 2014; López-Sanz et al., 2016). Therefore, alpha band RP may be a promising stable neurophysiological indicator for MI performance.

From on-task analysis, changes of RP were also found to correlate with performance. Subjects with higher performance seemed to have a larger decrease in alpha band ΔRP and a larger increase in theta and gamma band ΔRP (see Figure 5). The decrease of the alpha band ΔRP is likely to be related to ERD, which may make MI tasks more distinguishable when ERD is larger. Notably, previous studies proved that ERD mainly focused on the bilateral motor cortex, whereas the results of ΔRP showed that SMA and PM also participate in the process of MI. The larger increase in theta and gamma band ΔRP was possibly affected by the decrease of total power due to the ERD. Besides, higher RP of theta band was considered to be associated with cognitive performance or memory consolidation (Finnigan and Robertson, 2011; Reiner et al., 2014), and low gamma RP was found to be significantly negatively correlated with the inattention score (Roh et al., 2016). Here, the larger increase in theta and gamma band ΔRP may represent more consistent MI patterns or higher attentional levels and thus result in a better performance.

To investigate the similarities and differences in performance variation over subjects and time, the RP factors were further analyzed across sessions in this research. Results showed a similar relationship between binary-class performance and ΔRP from theta, alpha, and gamma bands, but no significant correlation was found in three-class performance (see Figure 6). Further analysis shows that the inter-subject correlations between ΔRP and performance are task-independent. However, from inter-session analysis, ΔRP was more likely to be correlated with the performance from tasks which contained idle task (see Table 1). This result suggests that RP factors from the pre-task resting state and the idle task may have a similarity and represent mental states that changed over time. This is further exemplified in the previous study that enhancing the alpha band during resting state by mindfulness training could improve BCI performance between MI and idle task rather than MI of individual hands (Stieger et al., 2020).

In this study, feedback was not applied to limit autonomic regulation when engaged in BCI tasks. Although subjects cannot see the feedback during the task, they were informed that the financial compensation was related to their offline performance to ensure a higher engagement. The averaged accuracy among subjects showed no significant performance increase across sessions (see Figure 2C). This result may suggest that without feedback, subjects could not regulate their brain to reach the expected output even over a relatively long time. In the literature, feedback has long been identified as an effective way for learning and training in BCI systems. Usually, the feedback indicates whether subjects have successfully achieved a task or not, which is unintuitive for subjects to learn to improve their control strategies (Jeunet et al., 2016). Recent studies on neurofeedback treatment gained extensive attention (Alkoby et al., 2018; Jeunet et al., 2018). Neurophysiological factors might be more appropriate instructions to help subjects learn to modulate brain signals. The preliminary findings have shown that neurofeedback of the alpha band could improve cognitive performance (Zoefel et al., 2011) and MI-BCI performance (Bamdadian et al., 2015). Another co-adaptive BCI training study showed that performance was associated with modulation of the alpha band power in subjects with larger improvement (Abu-Rmileh et al., 2019). In this study, RP factors have been proposed as a reliable neurophysiological indicator of MI performance. Therefore, future studies on BCI neurofeedback or neural training paradigms using neurophysiological factors such as RP are recommended to help subjects modulate their brain oscillations more effectively and improve their BCI performance, so that more subjects may benefit from the advantages of BCI interventions.

A limitation of this study is that only power features of rhythms were exploited, and other analysis aspects such as brain connectivity features should be involved to better understand the mechanisms underlying the BCI performance variation. Besides, the beta band is thought to be an important rhythm in MI tasks. However, in this study, no significant change was found in beta band RP, which may be diminished due to the RP calculation method. Moreover, experiments with larger sample size and over the longer term should be undertaken in further studies.

## Conclusion

Neurophysiological signals have been considered a promising target to understand how BCI performance varies. In this paper, the results indicate that changes in relative EEG rhythms power significantly correlated with variation in MI performance across time and subjects. Specifically, the pre-task alpha band RP was found to positively correlate with performance and was proposed as a stable neurophysiological indicator. During the task, results showed that the alpha band ΔRP was negatively correlated with performance, whereas theta and gamma band ΔRP were positively correlated with performance. From intra-subject analysis, a similar relationship between performance and ΔRP factors was found. This is the first study of performance variation which examines from pre-task to task and both across time and subjects. The findings of this study complement those of earlier studies and could inform future experiments to investigate the effects of neurophysiological factors on building BCI systems with neurofeedback.

## Data Availability Statement

The raw data supporting the conclusions of this article will be made available by the authors, without undue reservation.

## Ethics Statement

The studies involving human participants were reviewed and approved by the Human Research Ethics Committee of The Second Affiliated Hospital of Zhejiang University School of Medicine. The participants provided their written informed consent to participate in this study.

## Author Contributions

QZ conducted the study and performed the data analysis. JL and YH assisted in setting up the system and performing the experiment. QZ and KX wrote the text of the manuscript. LY, YW, and KX supervised the study and proofread the manuscript. All authors read and approved the final article.

## Conflict of Interest

The authors declare that the research was conducted in the absence of any commercial or financial relationships that could be construed as a potential conflict of interest.

## Publisher’s Note

All claims expressed in this article are solely those of the authors and do not necessarily represent those of their affiliated organizations, or those of the publisher, the editors and the reviewers. Any product that may be evaluated in this article, or claim that may be made by its manufacturer, is not guaranteed or endorsed by the publisher.
