# Corrigendum: Relative Power Correlates With the Decoding Performance of Motor Imagery Both Across Time and Subjects

**DOI:** 10.3389/fnhum.2022.977379

**Published:** 2022-07-19

**Authors:** Qing Zhou, Jiafan Lin, Lin Yao, Yueming Wang, Yan Han, Kedi Xu

**Affiliations:** ^1^Key Laboratory of Biomedical Engineering of Education Ministry, Department of Biomedical Engineering, Qiushi Academy for Advanced Studies, Zhejiang University, Hangzhou, China; ^2^Zhejiang Lab, Hangzhou, China; ^3^Frontiers Science Center for Brain and Brain-Machine Integration, Zhejiang University, Hangzhou, China; ^4^The College of Computer Science and Technology, Zhejiang University, Hangzhou, China; ^5^Zhejiang Key Laboratory of Neuroelectronics and Brain Computer Interface Technology, Hangzhou, China

**Keywords:** relative power, brain rhythms, motor imagery, performance variation, electroencephalogram

In the published article, there was an error in affiliations 1 and 2 as published. Instead of “^1^ Zhejiang Lab, Hangzhou, China, ^2^ Key Laboratory of Biomedical Engineering of Education Ministry, Department of Biomedical Engineering, Qiushi Academy for Advanced Studies, Zhejiang University, Hangzhou, China,” it should be “^1^ Key Laboratory of Biomedical Engineering of Education Ministry, Department of Biomedical Engineering, Qiushi Academy for Advanced Studies, Zhejiang University, Hangzhou, China, ^2^ Zhejiang Lab, Hangzhou, China.”

The authors apologize for this error and state that this does not change the scientific conclusions of the article in any way. The original article has been updated.

## Publisher's note

All claims expressed in this article are solely those of the authors and do not necessarily represent those of their affiliated organizations, or those of the publisher, the editors and the reviewers. Any product that may be evaluated in this article, or claim that may be made by its manufacturer, is not guaranteed or endorsed by the publisher.

